# Wideband tunable microwave signal generation in a silicon-micro-ring-based optoelectronic oscillator

**DOI:** 10.1038/s41598-020-63414-9

**Published:** 2020-04-24

**Authors:** Phuong T. Do, Carlos Alonso-Ramos, Xavier Le Roux, Isabelle Ledoux, Bernard Journet, Eric Cassan

**Affiliations:** 1 Université Paris-Saclay, ENS Paris-Saclay, CNRS, Lumin, 4, avenue des Sciences, 91190 Gif-sur-Yvette, France; 2 Université Paris-Saclay, CNRS, Centre de Nanosciences et de Nanotechnologies, 10 Boulevard Thomas Gobert, 91120 Palaiseau, France

**Keywords:** Nanoscience and technology, Optics and photonics

## Abstract

Si photonics has an immense potential for the development of compact and low-loss opto-electronic oscillators (OEO), with applications in radar and wireless communications. However, current Si OEO have shown a limited performance. Si OEO relying on direct conversion of intensity modulated signals into the microwave domain yield a limited tunability. Wider tunability has been shown by indirect phase-modulation to intensity-modulation conversion. However, the reported tuning range is lower than 4 GHz. Here, we propose a new approach enabling Si OEOs with wide tunability and direct intensity-modulation to microwave conversion. The microwave signal is created by the beating between an optical source and single sideband modulation signal, selected by an add-drop ring resonator working as an optical bandpass filter. The tunability is achieved by changing the wavelength spacing between the optical source and a resonance peak of the resonator. Based on this concept, we experimentally demonstrate microwave signal generation between 6 GHz and 18 GHz, the widest range for a Si-micro-ring-based OEO. Moreover, preliminary results indicate that the proposed Si OEO provides precise refractive index monitoring, with a sensitivity of 94350 GHz/RIU and a potential limit of detection of only 10^−8^ RIU, opening a new route for the implementation of high-performance Si photonic sensors.

## Introduction

The generation of broadband and low noise microwave and millimeter wave signals is important for many applications, including among others, radars^1,2^ wireless communications^3^, optical signal processing^4^, warfare systems^5,6^ and modern instrumentation^7^. The optoelectronic oscillator (OEO) is a particularly interesting solution to generate microwave and millimeter signals due to its capability to provide direct synthesis of spectrally pure and wideband tunable signals^8,9^. A classical OEO has a fundamentally multi-mode behavior^10^, with mode spacing associated with the km-long optical fiber delay lines used inside the closed-loop system. To select the desired oscillation mode, a microwave filter with high quality factor (Q_*RF*_) is typically included inside the closed path^10,11^. To achieve variable frequency generation, this microwave filter needs to be tunable. However, the realization of microwave filter with high Q_*RF*_ and wide frequency tunability is practically hard to realize, especially for high operation frequencies^12^. In contrast, microwave photonics (MWP) is a promising alternative solution to overcome this limitation, allowing reconfigurable microwave signal generation in OEO with a wide tuning range^11–13^. In addition, the progress of integrated microwave photonics (IMWP)^14^ provides now a solid framework for the full OEO integration. Several approaches have been recently demonstrated^15–17^. M. Merklein *et al*., in^15^ reported the generation of ultrawide frequency tunable signals up to 40 GHz by using OEO based on stimulated Brillouin scattering (SBS). However, this approach requires harnessing light-sound interactions on chip, based on non-standard chalcogenide materials and the use of two lasers. In^16^, an integrated optoelectronic oscillator based on InP was investigated, but the frequency tunability was limited to only 20 MHz. Remarkable low phase noise of −110 dBc/Hz and −130 dBc/Hz at 10 kHz and 10 MHz offset frequency has been demonstrated for OEO based on nonlinear frequency comb generation in silicon nitride micro-resonators^18^. However, the frequency of the microwave signal is determined by the free-spectral range (FSR) of the ring. Once the ring is fabricated, the FSR can only be slightly shifted, substantially limiting the tunability of this approach. On the other hand, the silicon on insulator (SOI) technology has been identified as a promising solution to implement ultra-compact and low-cost OEO, which could be fabricated using already existing large volume fabrication facilities. The unique potential of Si to integrate photonic and electronic functionalities within a single chip, together with the availability of high-performance key building blocks, e.g. all-Si modulators^19,20^ and Ge on Si photodetectors^21,22^ and even silicon RF power amplifiers^23^, make Si an ideal candidate for the development of high-performance OEOs. For instance, remarkable low phase noise has been recently demonstrated for an OEO that implements all electronic and photonic building blocks with silicon technology, expect for the laser^24^. Even if the reported tuning range of this OEO is of only a few MHz, these results illustrate the great potential of the silicon technology for the implementation of high-performance OEOs.

**Figure 1:**
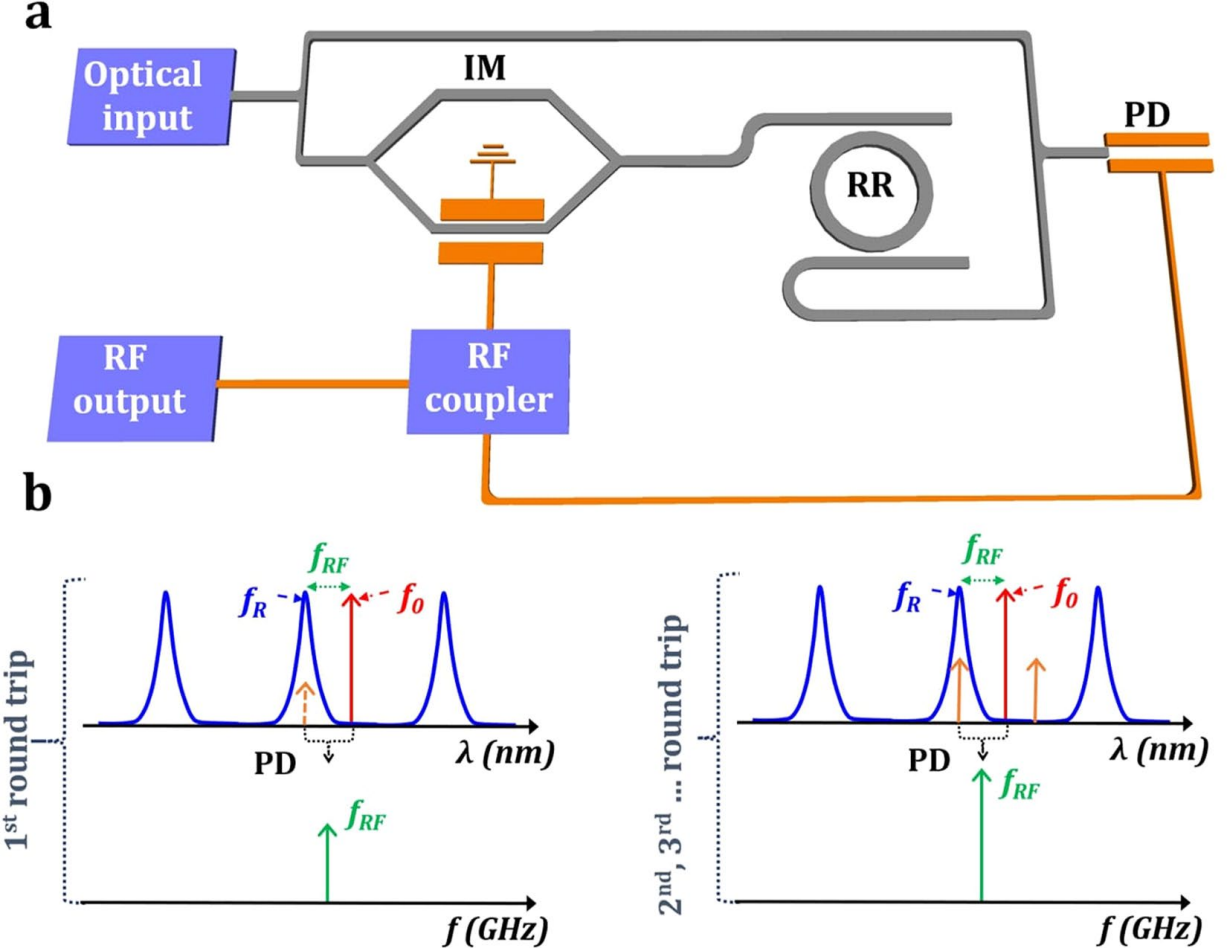
(**a**) Schematic of the proposed OEO structure and (**b**) Principle of operation of the proposed tunable OEO. In (**a**), IM: Intensity modulator, RR: Ring resonator and PD: Photo-detector. In (**b**), the red curve corresponds to the optical carrier (or laser source frequency); the orange curve illustrates the sideband lobes of the modulated signal, the blue curve indicates the optical transfer function of the RR and the green one represents the generated RF frequency f_*RF*_.

However, the scarce demonstrations of Si-based OEOs showed a limited performance in terms of tunability. Direct conversion of intensity-modulated signals into the microwave domain has been shown based on quadratic detection of two successive transmission lines in the drop-port of the ring^25^. The microwave frequency is determined by the FSR of the ring, limiting its tunability. In addition, microwave signal generation requires few-millimeters long ring resonators, which are difficult to implement. Microwave generation has also been demonstrated in Si-micro-resonator-based OEO, implementing indirect phase-modulation to intensity-modulation conversion. In this case, the notch filtering is performed by a micro-disk in operating in an all-pass configuration. This approach provided a limited tunability range between 3 and 6.8 GHz^17^. Here, we propose a new approach for the implementation of Si-micro-ring OEO that enables wideband tunability in the microwave signal generation, exploiting direct intensity-modulation to microwave conversion. As schematically shown in Fig. 1a, the laser source is split in two paths. One path comprises an intensity modulator and an add-drop ring resonator (RR). The other path goes directly to the photodetector. The oscillation signal is created by the direct translation of the intensity modulation into the microwave domain, provided by the beating between the optical source (direct path) and one of the sideband lobes generated by the intensity modulator (path with intensity modulator and RR). This sideband lobe is selected by one transmission line of the silicon add-drop RR, that serves as optical bandpass filter. The frequency of the generated microwave signal is determined by the wavelength separation between the laser source and the resonance of the RR. By using only one of the transmission lines of the RR, we substantially relax the requirements on the free-spectral-range of the ring, while providing flexible tuning. We experimentally show that by tuning the wavelength of the source, the microwave frequency generated by the OEO can be tuned between 5.9 GHz and 18.2 GHz. This is, to the best of our knowledge, the widest tunability range reported for a Si-micro-ring-based OEO. A phase noise near −110 dBc/Hz at the offset frequency of 1 MHz, comparable with state-of-the-art photonic tunable OEO^16,17^, is measured for different oscillation frequencies along the 12 GHz tuning range. Concurrently, the proposed OEO performs a precise translation of the laser-to-RR wavelength separation into the microwave domain, where it can be precisely measured. Then, if the laser wavelength is fixed, monitoring of the microwave frequency shifts provides accurate information of the variations in the resonance wavelength of the RR, which can be related to variations in the refractive index. This way, by exploiting the improved spectral resolution in the microwave domain, the proposed OEO can also serve as a high-performance refractive index sensor. Preliminary experimental results show a sensitivity of 94350 GHz/RIU, i.e. a 40-fold improvement compared to previously reported microwave-photonic silicon refractive index sensors^26^. We estimated the achievable limit of detection (LOD) from the phase noise measurements, obtaining a remarkably low value of 10^−8^ RIU. These results illustrate the potential of this approach for the implementation of high-performance Si sensors, e.g. for lab-on-a-chip biosensing applications^27^.

## Results

### Principle of operation

In the proposed tunable OEO configuration, shown in Fig. 1, the optical signal coming from the laser light source (frequency f_0_) is separated into two arms. One is connected directly to the photodetector (PD), while the other feeds an intensity modulator (IM) followed by a silicon ring resonator (RR) in add-drop configuration. In this scheme, the input signal of the PD always comprises a part of the un-modulated laser light beam. At the initial stage, the modulator output signal grows seeded by the white noise existing inside the loop. If one modulated signal (f_*R*_) can go through the optical transfer function of the resonator, it can then be combined with the optical carrier (f_0_) in the PD generating a beating of frequency f_*b*_. If the distance between the optical carrier (f_0_) and the signal at f_*R*_ falls within the working range of the loop, the generated beating signal can be converted to the RF domain f_*RF*_ (f_*RF*_ = f_*b*_ = |f_0_ − f_*R*_|). At the second round-trip of the loop, the generated RF signal is sent back to the modulator. At this stage, only one single side-band modulation signal can match the RR resonance peak at frequency f_*R*_ (see Fig. 1b). The RR now serves as an optical bandpass filter, selecting only one sideband lobe of the modulated signal. The signal goes to the PD at the second-round trip of the loop, creating again an RF signal with frequency f_*RF*_. After this point, the loop oscillates with an oscillation frequency of f_*RF*_.

The main idea behind this approach is to control the frequency of the microwave signal by the wavelength spacing between the laser source and the resonance wavelength of the resonator. Since this spacing can be changed either by sweeping the wavelength of the laser or by shifting the resonance peak of the RR, this approach yields a simple tunability mechanism. The laser wavelength can be swept using a tunable laser. By monitoring the microwave signal generated, it is possible to choose the proper wavelength value yielding the desired microwave frequency. In a similar fashion, the resonance peak of the RR can be tuned, for example exploiting the thermo-optic effect to change the effective index of the waveguide, thereby shifting the resonance wavelength^28^.

### Demonstration of the proposed tunable optoelectronic oscillator

Even if micro-disk and micro-sphere resonators could yield higher quality factors, for the implementation of the optical bandpass filter in our OEO, we have chosen a micro-ring resonator. Micro-spheres are difficult to integrate on chip together with other photonic components. On the other hand, high-Q micro-disk typically rely on large bending radii that result in highly multimode behavior, which could distort the response of the OEO, e.g. by mode hopping due to the existence of several resonances within the working range of the OEO. Conversely, micro-ring resonators can be made single-mode, regardless of the radius, just by ensuring the waveguide is single mode.

**Figure 2:**
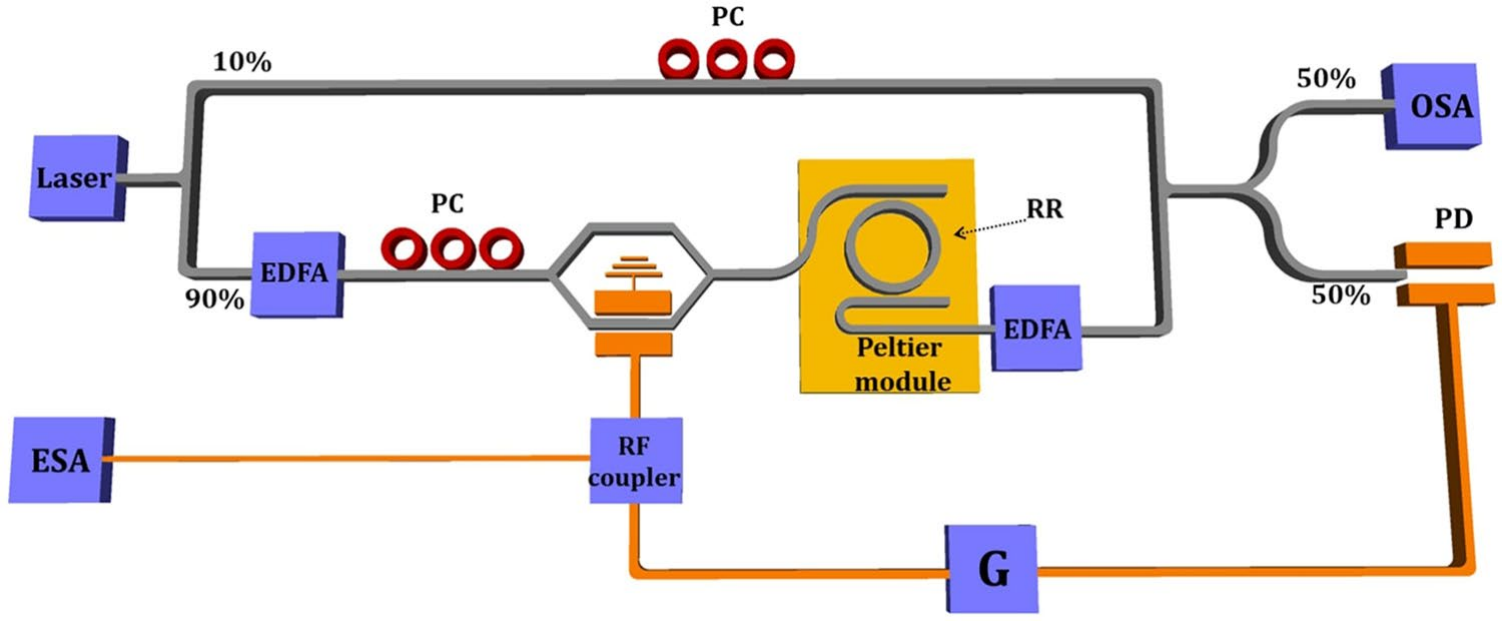
Experimental setup employed for the demonstration of the proposed tunable OEO. EDFA: Erbium doped amplifier, PC: Polarization controller, OSA: Optical spectrum analyzer, G: RF amplifier and ESA: Electric spectrum analyzer.

To demonstrate the proposed operation principle, we used an integrated Si add-drop RR and external intensity modulator, photodetector and microwave circuitry (see Fig. 2). It should be pointed out that all external building blocks have already been demonstrated in the silicon technology. Thus, monolithic integration of the complete OEO is technologically feasible. Nevertheless, the proposed scheme serves as a demonstrator of the principle, while providing a simple and flexible implementation, as different Si ring resonators can be tested using the same global circuit.

The Q in the add-drop ring resonator is one of the key parameters determining the performance of the proposed OEO. Higher Q yields better selectivity of the optical filter, that will determine the purity and stability of the microwave signal generated. The ring resonator was implemented on a standard SOI technology with a 220 nm thick Si thin film on top of a 3 *μ*m buried oxide layer. We optimized the ring to operate in transverse-magnetic (TM) polarization, thereby minimizing the detrimental effect of sidewall roughness in propagation loss.

**Figure 3:**
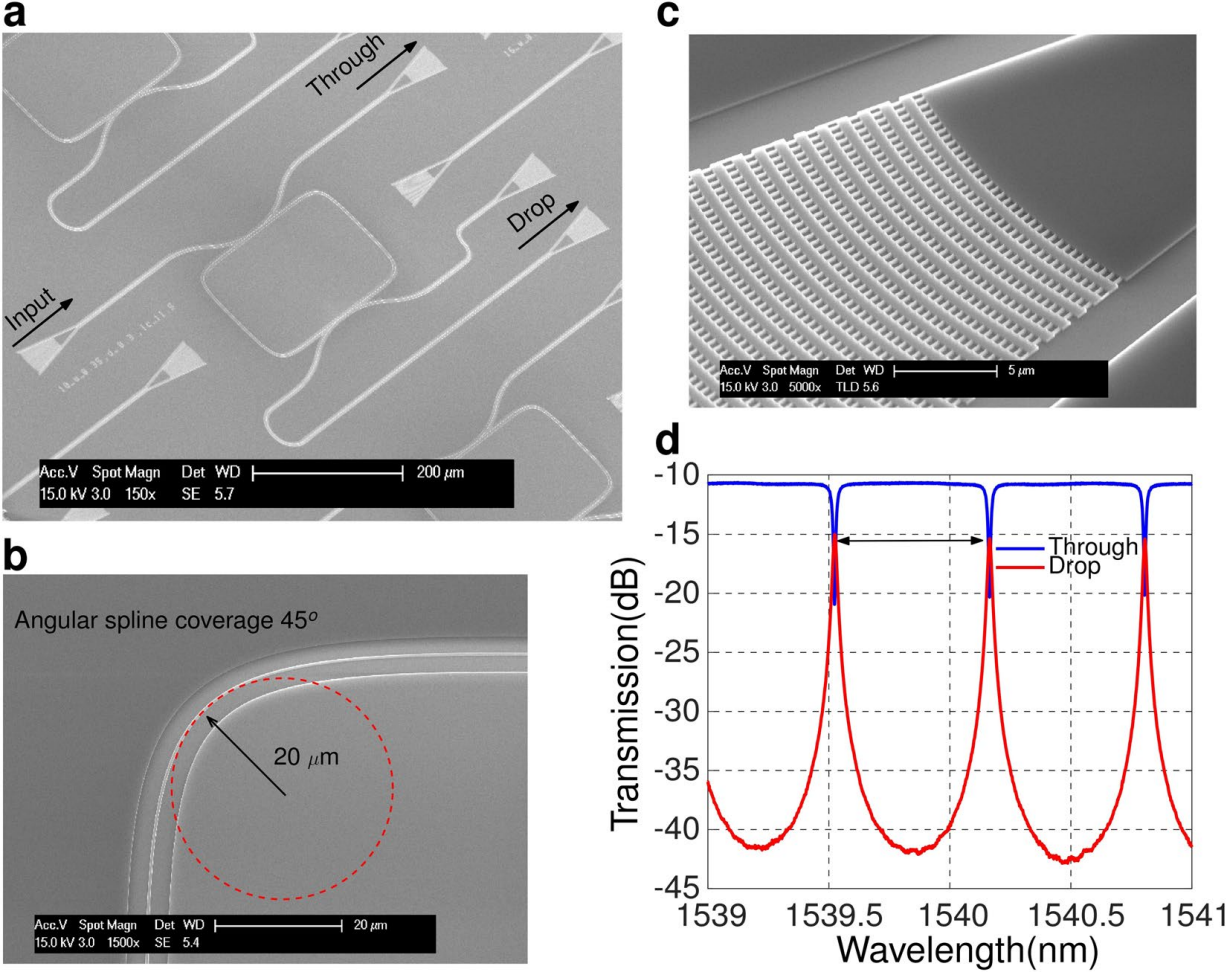
Scanning electron microscope images of: (**a**) complete add-drop RR, (**b**) adiabatically bended waveguide and (**c**) fiber-chip grating coupler. (**d**) Optical transmission of the silicon RR (coupling gap of 300 nm and coupling length of 4.5 *μ*m).

Figure 3a shows an electron microscope image of the add-drop ring resonator. Light is injected into the resonator through the input port and collected from the through or drop ports. A detailed view of the fiber-chip grating couplers used to inject and extract the light is shown in Fig. 3c. A 450 nm wide strip waveguide was chosen to ensure single mode operation near 1.54 *μ*m wavelength, with a resonator length L of 1 mm. In the RR, adiabatic bends^29^ were implemented to reduce losses coming from the mode mismatch at the transition between straight and circular bend waveguides. Spline bend was made with 20 *μ*m radius and angular spline coverage angle at 45° (see Fig. 3b). Figure 3d shows the measured transmission spectra (see Methods) of both through and drop ports of the RR with 300 nm coupling gap and 4.5 *μ*m coupling length. An optical FSR_*λ*_ of 640 pm was obtained, corresponding to a microwave free-spectral range of FSR_*fre*_ ≈ 77 GHz. The optical quality factor of the ring resonator was Q_*opt*_ ~ 8.1 × 10^4^ (obtained by Lorentzian fitting of the resonance peaks).

Figure 2 shows the experimental setup used to demonstrate the proposed OEO approach. We used a 90/10 optical splitter to separate the light source coming from a CW tunable laser (Yenista TUNIS-T100S). 90% of the optical power was sent to an Erbium doped amplifier (EDFA) followed by the intensity modulator, the silicon RR and a second EDFA. After that, a 50/50 optical combiner was used to collect the signal from the output of a second EDFA and the optical power source signal (see Fig. 2). Polarization controllers (PC) were used in the upper arm of the splitter to match the polarization of the laser source and the signal at the output of the second EDFA. At the output of the optical combiner, one arm was connected to an optical spectrum analyzer (OSA) to monitor the laser or resonance wavelength, while the other arm was connected to the PD. The final setup included an RF amplifier, a 90:10 RF coupler and an electrical spectrum analyzer (ESA).

**Figure 4:**
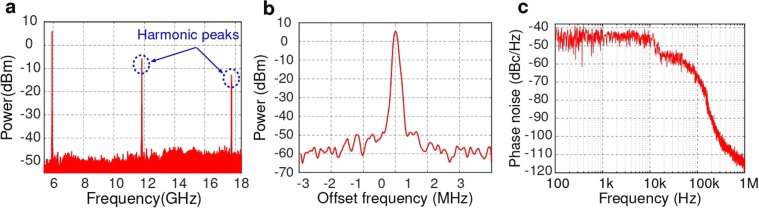
(**a**) Oscillation spectrum of the generated signal based on our proposed approach, (**b**) The zoom-in viewed and (**c**) The phase noise characteristic of the created signal.

During the experiments, the resonance frequency of the RR f_*R*_ was first monitored using an OSA. Then, the oscillation frequency is generated by placing the laser wavelength (frequency f_0_) close to a resonance peak of the RR. Figure 4a illustrates the electrical spectrum of the generated microwave signal within a frequency span of 13.5 GHz and with a resolution bandwidth of 200 kHz, showing an oscillation frequency at 5.9 GHz. In addition, higher-order harmonic peaks at 11.8 GHz and 17.7 GHz were also observed, caused by the nonlinearity in the OEO loop^17^. The zoomed-in view of the 5.9 GHz signal with a frequency span of 6 MHz and a resolution bandwidth of 2.2 kHz is shown in Fig. 4b. The microwave signal exhibits a high signal to noise ratio of 60 dB with the linewidth around 120 kHz. To evaluate the stability and the quality of the generated signal, we measured its phase noise with an electrical RF analyzer (Agilent E4446A). The measured phase noise, shown in Fig. 4c. It is worth mentioning that the relatively high and flat phase noise level close to the carrier frequency, up to 100 KHz, is produced by wavelength fluctuations of our laser^25^. The effect of laser fluctuations is negligible for higher frequencies. There, our OEO yields the measured phase noise of −115 dBc/Hz at 1 MHz offset frequency from the carrier. This result is comparable with the phase noise recently reported in photonic OEO implemented in silicon^17^.

**Figure 5:**
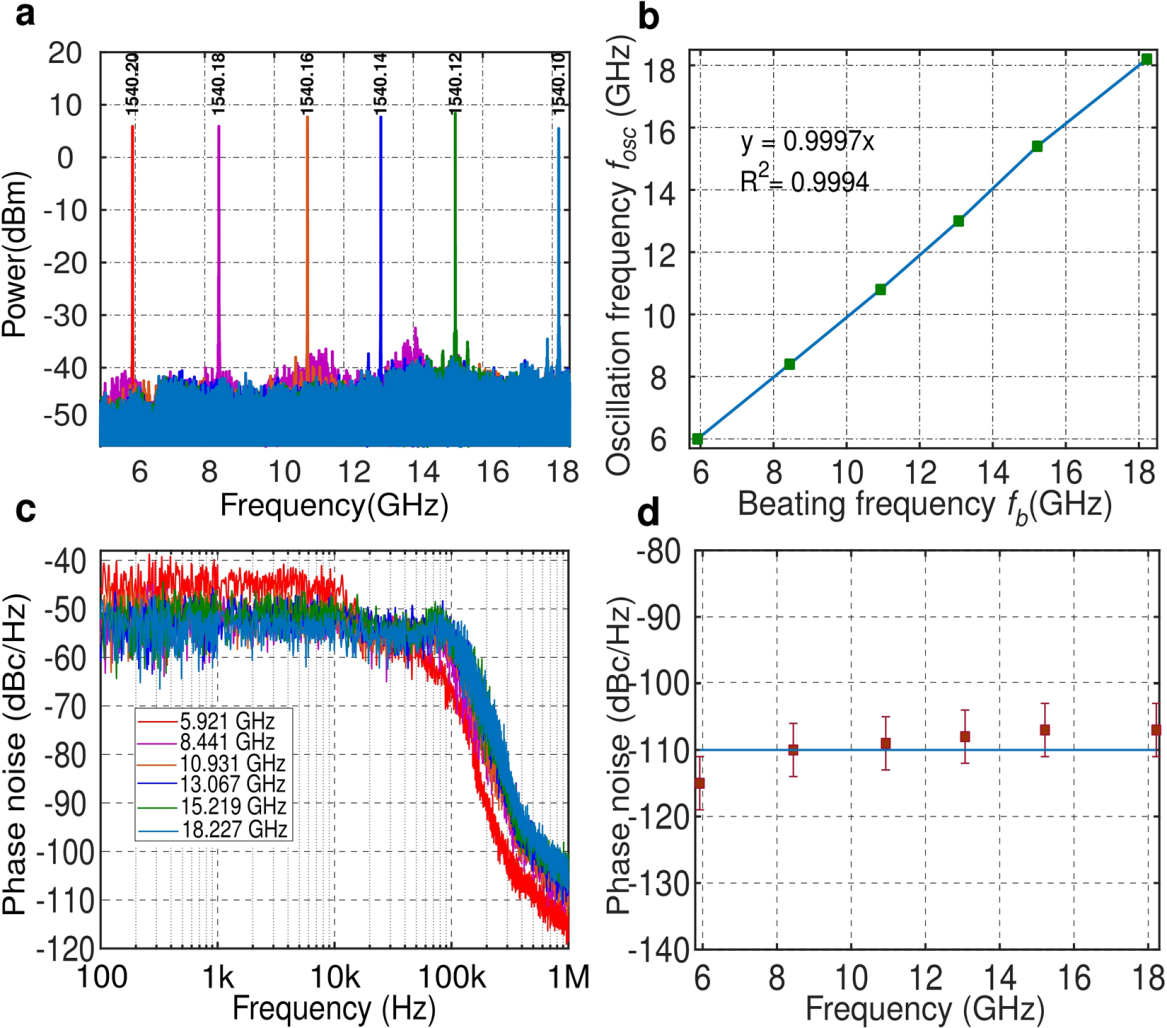
(**a**) Oscillation frequency generated with different laser wavelengths, (**b**) Plot of the oscillation frequency depending on the beating frequency. f_*b*_ = |f_0_ − f_*R*_|, (**c**) Phase noise characteristic for differences generated signals and (**d**) Observed phase noise level at 1 MHz offset frequency from carrier.

In order to demonstrate the wide tunability of the proposed approach, we swept the laser wavelength while keeping the resonance peak unchanged. To do so, the Si chip was placed on a Peltier module to stabilize the temperature, thereby preventing resonant wavelength shifts produced by thermal drifts. The resonance wavelength of the RR at 1540.25 nm was first measured with the OSA. Figure 5a plots the fundamental tone of the oscillation spectrum obtained by sweeping the laser wavelength between 1540.10 nm and 1540.20 nm. These experimental results demonstrate an unprecedentedly wide frequency tunability for a Si-micro-resonator-based OEO, ranging from 5.9 GHz to 18.2 GHz. The tuning range is limited here by the bandwidth of the microwave amplifier used inside the loop. Along the 12 GHz spectrum, a power fluctuation in the 1-~3 dB range has been observed. This fluctuation could be attributed to two main effects: i) mechanical variations of the system that change the fiber-chip coupling loss, ii) fluctuations of the supply current applied to the EDFA, as the pump current variation changes of the overall loop gain^11^. Nevertheless, this variation range is slightly smaller than what was reported in^17^. Note that, the minimum frequency tuning step of the OEO is determined by the precision in controlling the wavelength of the laser and the temperature of the ring resonator. For example, working at a fixed temperature, the minimum tuning step of the OEO is ~125 MHz, determined by the 1 pm resolution of the laser source we are currently using. Higher resolution could be achieved with a laser source enabling narrower wavelength steps.

From the frequency of the laser and resonance wavelength, we calculated the beating frequency, i.e. f_*b*_ = |f_0_ − f_*R*_|. The evolution of the oscillation signal (f_*osc*_) as a function of the beating frequency is shown in Fig. 5b. The oscillation frequency clearly follows the beating frequency, showing a nearly perfect linear evolution with the modification of the laser frequency separation from the RR resonance frequency (regression coefficient ≈ 0.9997). In this case the RR resonance frequency is smaller than the laser one, which explains why the oscillation frequency increases with decreasing laser frequency.

We have measured the phase noise of the proposed tunable OEO, for all the generated signals (see Fig. 5c). As has been explained, the phase noise at frequency offsets below 100 kHz is governed by the random wavelength fluctuations of the laser, resulting in unstable signals that are difficult to compare. For frequency offsets beyond 100 kHz, the phase noise is governed by the actual response of the OEO, exhibiting a phase noise variation below 7 dB in all the frequency span between 5.9 GHz and 18.2 GHz. Figure 5d represents the noise level at 1 MHz offset frequency from the carrier, obtained from the measurements in Fig. 5c. The phase noise of the generated microwave signals remains close to −110 dBc/Hz at the offset frequency of 1 MHz, which emphasizes a key advantage of such an OEO to have a constant phase noise level with the increase in oscillation frequency^30^.

### The OEO as refractive index sensor

**Figure 6:**
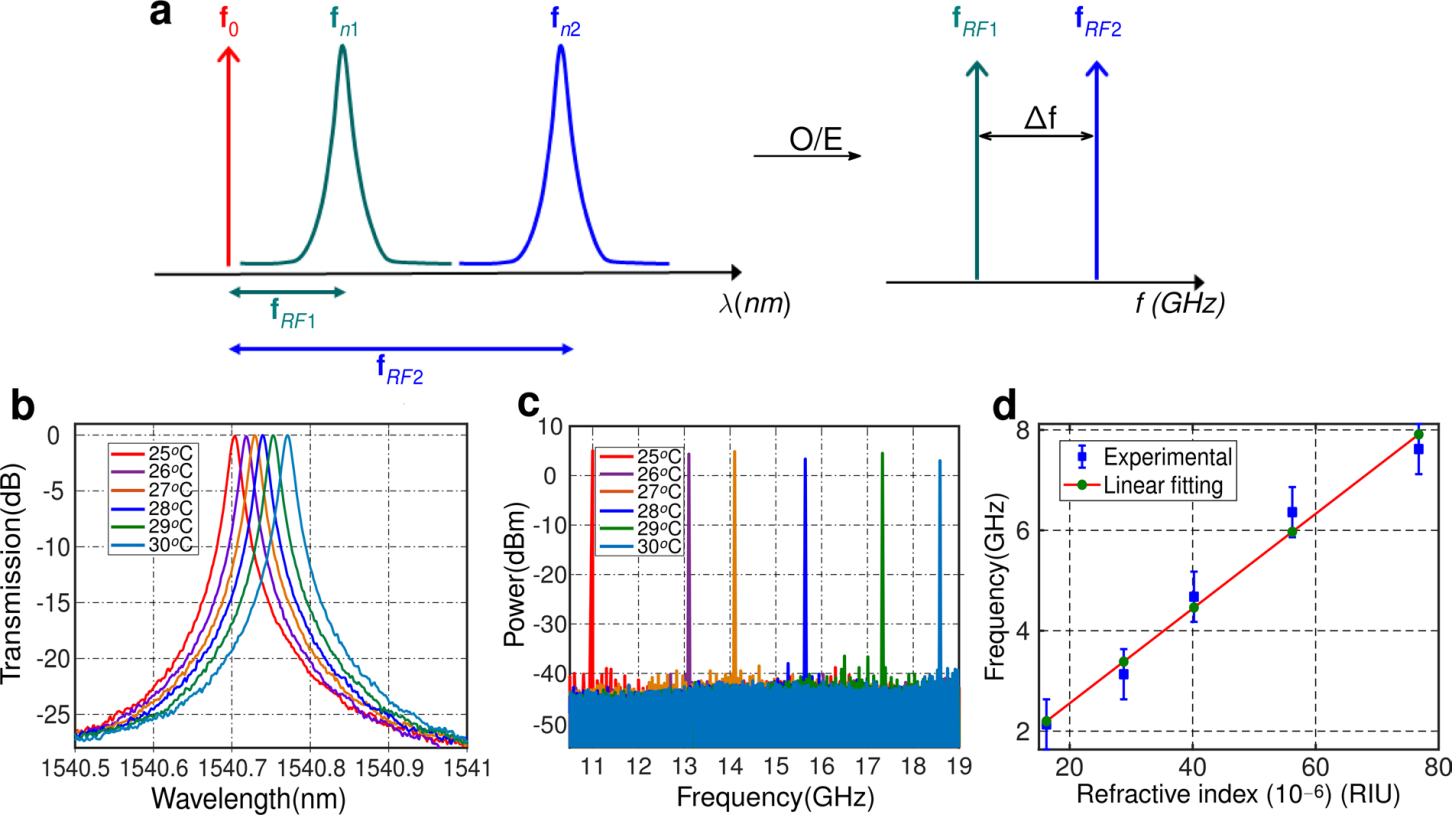
(**a**) Principle of operation of the sensing system based on the proposed OEO configuration. f_0_: Optical carrier (or laser source frequency), f_*n*1_ and f_*n*2_: resonances wavelength associated with cladding index n_1_ and n_2_,respectively, f_*RF*1_ and f_*RF*2_: generated oscillation frequency associated with cladding index n_1_ and n_2_, respectively, Δ*f* = |f_*RF*1_ − f_*RF*2_|. (**b**) Resonance wavelength, (**c**) Oscillation frequency simultaneously collected for each setting temperature point in the Peltier module and (**d**) Calculation of the oscillation change depending on refractive index variation.

The oscillation frequency of the proposed OEO is determined by the relative distance between the laser and the ring resonance. The ring resonance depends on the refractive index of the waveguide and its environment. Thus, by fixing the laser wavelength and monitoring the shifts in the microwave oscillation frequency, it is possible to precisely estimate the variations in the optical index^31,32^. This sensing mechanism is schematically described in Fig. 6a. At the initial stage, the laser and the ring resonance are separated by a given distance, generating a microwave signal oscillation with frequency ${f}_{RF1}$. Any variation in the refractive index of the waveguide or its environment shifts the ring resonance, producing a proportional shift in the frequency of the microwave oscillation, from ${f}_{RF1}$ to ${f}_{RF2}$. As a simple means to implement this index change, we changed the sample temperature with a Peltier module. The temperature variation of the ring resonator shifts its resonance wavelength. We measured this wavelength shift in the optical domain (see Fig. 6b) and extracted the index variation. At the same time, we monitored the variations in the oscillator frequency (see Fig. 6c). Then, as shown in Fig. 6d, we could plot the oscillation frequency shift as a function of the refractive index change, obtaining a linear relation.

In order to get a stable local temperature over the RR sample region, experiments only started 5 minutes after setting the desired temperature point in the Peltier module (in the range from 25 °C to 30 °C with 1 °C step size). The RR was first characterized in the optical domain. Right after the optical characterization, we characterized the frequency generated by the OEO. We used a highly stable distributed feedback (DFB) diode laser (model 1905 LMI) with wavelength near 1.54 *μ*m. Optical and microwave spectra, measured while scanning the temperature with the Peltier module between 25 °C and 30 °C, are shown in Fig. 6b,c, respectively. The resonance wavelength shifts towards longer wavelengths with increasing temperature, resulting in a microwave frequency increase from 11 GHz to 18.5 GHz. These results are in good agreement with previous theoretical and experimental studies made for SOI ring resonators^28,31^. For example, in^31^ the RF spectra is shown for OEO frequency changing between 10 GHz and 18 GHz. However, no study of phase noise is performed in^31^, hindering the evaluation of this OEO as a microwave photonic sensor.

The variation in the optical index was estimated from the measured optical spectra as Δn_*eff*_ = n_*eff*_.Δ*λ*/*λ*^33^. We calculated the waveguide refractive index n_*eff*_ with a vectorial mode solver. We took the first detected resonance wavelength and oscillation frequency as the reference point. Then we estimated the refractive index change from the wavelength resonance shift. The oscillation frequency shift as a function of the estimated refractive index change is plotted in Fig. 6c. We obtain a slope of 94350 GHz/RIU (linear fitting). This value is 40 times better than previously reported microwave-photonic silicon refractive index sensors using a SOI resonator device to detect frequency changes produced by cladding refractive index change^26^. Note that both, our work and that reported in^26^ realize on integrated Si ring resonator and external components. On the other hand, the sensor presented in^26^ implements a noise reduction algorithm based on phase-only filtering and uses two etching steps for the fabrication of the Si micro-resonator, one full-etch for the interconnection waveguides and one shallow etch for the disk resonator. We do not apply any post-processing filtering technique and fabricate our device with a single etch step. Our approach exhibits a stable phase noise level near −110 dBc/Hz at 1 MHz offset frequency from the carrier, allowing a system resolution of 1 MHz. From this phase noise value, we estimate a limit of detection (LOD) as low as 10^−8^ RIU.

## Discussion

In summary, we have proposed and experimentally demonstrated a new approach for the implementation of widely tunable Si-micro-resonator-based OEO. Previously reported Si OEO relied on direct conversion of intensity modulation to the microwave domain, with limited tunability, or indirect phase-modulation to intensity modulation conversion. Here, we show a direct conversion scheme providing wide tunability. In the proposed scheme, the microwave signal is created by the beating between a laser light source and a single sideband modulation signal selected by an add-drop ring resonator working as an optical bandpass filter. The microwave frequency is determined by the wavelength separation between the source and the ring resonance, providing simple tunability by sweeping the laser wavelength. Capitalizing on this concept, we demonstrate microwave signal generation between 5.9 GHz and 18.2 GHz, only limited here is the bandwidth of the employed RF amplifier. This is the widest microwave generation span reported for a Si-based OEO. Additionally, a low phase noise level close −110 dBc/Hz at 1 MHz offset frequency is achieved for all microwave frequencies, illustrating the potential of the approach for the generation of stable high oscillation frequency signals. As mentioned above, the poor phase noise levels at frequency offset close to the carrier mainly arise from the laser wavelength fluctuations. Introducing a DFB laser or feedback control loop could lower these effects^16^. Indeed, by using a laser with a better stability, a ~40 dB of noise level reduction at 10 kHz offset frequency was previously observed^34^. Moreover, the OEO phase noise could effectively be improved by increasing the quality factor of the micro-ring resonator. This could be achieved by reducing the waveguide propagation loss by thermal oxidation of the Si waveguides to reduce roughness and thus the propagation loss level^35^. Based on experimental characterization of propagation loss in state-of-the-art silicon waveguides, we could target more than a ten-fold increase of the resonator Q. Then, following the Leeson’s model^36^, we could expect phase noise reduction of the OEO exceeding 10 dB. Thus, the phase noise of the proposed system could reach a level of less than −100 dBc/Hz at 10 kHz offset from carrier, which level is compatible with real applications^37^. Further minimization in phase noise property of the loop could be accomplished with a full integration of the OEO system on a single chip. Furthermore, we extended this approach for refractive index sensing application, harnessing high spectral resolution in the microwave domain. We have measured a sensitivity of 94350 GHz/RIU, 40 times better than state-of-the-art Si counterparts microwave photonic silicon refractive index sensor^26^ and have estimated a potential limit of detection as low as 10^−8^ RIU for an interrogation speed of 1 MHz. We believe that the approach proposed here will expedite the development of a new generation of high-performance Si OEO with an immense potential for a plethora of applications, including, radar, wireless communications, optical signal processing, warfare systems and lab-on-a-chip biosensing.

## Methods

### Device fabrication and experimental characterization

Fabrication started from a SOI wafer with a 220 nm thick Si thin film on top of a 3 *μ*m buried oxide layer. The patterns were lithographically defined in a 100 nm ZEP-520A photoresist by using e-beam lithography. After lithography, the patterns were transferred using ICP etching with SF_6_ and C_4_F_8_ gases. Following the waveguide fabrication, a 2 *μ*m thick PMMA layer was deposited over the chip surface for protection. No additional post processing was done.

For the optical characterization of the ring resonators, a tunable laser was coupled to the input waveguide through an input grating coupler with a properly adjusted coupling angle and extracted the same way from an output grating. The grating couplers were optimized for TM polarization, yielding a fiber to fiber optical transmission of −10.5 dB at 1540 nm wavelength. A polarization controller (PC) was used to set a proper polarization at the input of the grating.

## Data Availability

The data that support the findings of this study are available from the corresponding author upon reasonable request.
